# *Taare Zameen Par* and dyslexic savants

**DOI:** 10.4103/0972-2327.53077

**Published:** 2009

**Authors:** Ambar Chakravarty

**Affiliations:** Department of Neurology, Vivekananda Institute of Medical Sciences, Kolkata, India

**Keywords:** Artistic talent, dyslexia, *Taare Zameen Par*

## Abstract

The film *Taare Zameen Par* (Stars upon the Ground) portrays the tormented life at school and at home of a child with dyslexia and his eventual success after his artistic talents are discovered by his art teacher at the boarding school. The film hints at a curious neurocognitive phenomenon of creativity in the midst of language disability, as exemplified in the lives of people like Leonardo da Vinci and Albert Einstein, both of whom demonstrated extraordinary creativity even though they were probably affected with developmental learning disorders. It has been hypothesized that a developmental delay in the dominant hemisphere most likely ‘disinhibits’ the nondominant parietal lobe, unmasking talents—artistic or otherwise—in some such individuals. It has been suggested that, in remedial training, children with learning disorders be encouraged to develop such hidden talents to full capacity, rather than be subjected to the usual overemphasis on the correction of the disturbed coded symbol operations.

## *Taare Zameen Par* and dyslexic savants

The film *Taare Zameen Par* (Stars upon the Ground), a Hindi movie released toward the end of 2007, was a big box-office hit. The script was written by Amole Gupta and Deepa Bhatia and the film was directed by the noted actor Amir Khan. The film portrays the life of a young boy, Ishan Awasthi (enacted by 8-year-old Darsheel Safary), who is dyslexic and cannot read or write; he leads a tormented life until he meets with his art teacher, Ramashankar Nikumbh (enacted by Amir Khan), who discovers his artistic talents. Neurological illnesses had been portrayed earlier in various movies. Over 62 movies from USA, Europe, and the Far East have featured epilepsy.[1] From India, *Samman*, an epilepsy support group at Mumbai and Dr. Debasish Chowdhury from Delhi have produced excellent short films featuring patients with epilepsy. Dyslexic characters have featured in a number of works of fiction. Notable works include Henry Winkler's Hank Zipper series of children's books and Jennifer Weiner's novel 'In Her Shoes,' which was adapted as a film in 2005. Taki Wartooth and Skwisagaar Skwigelf both from the TV show ‘Netaocalypse’ claim to be dyslexic, as they cannot read music. ‘Shooting Fish’ features Dylan, a dyslexic conman, who makes his living using confidence tricks to gain money from rich people. He justifies his lifestyle by claiming that he is unable to get a job because of his dyslexia. Additionally, there are three episodes of the ‘The Cosby Show’ which focus on dyslexia, all three having Theo as one of the major characters in the plot. George Lopez from the eponymous TV show has dyslexia (source: http://www.wikepedia.com).

The present article focuses on the neural mechanisms of dyslexia, as well as the creativity and artistic talent that is seen in subjects with dyslexia, with special reference to the portrayal of one such case in *Taare Zameen Par*.

## Learning disabilities – An overview

Learning refers to the highest and most complex cognitive function of the brain and it should be of no surprise that many children (as many as 5-10% worldwide) have problems acquiring the basics of reading, writing, and mathematics. Many children in first and second grade just need some more time to acquire these basic skills. These temporary learning problems are frequent and reflect normal maturational variability. At the end of the continuous spectrum (and hence an arbitrary cutoff is to be used), however, there are children who really have fundamental and persistent learning problems. They are learning disabled.[2] A learning disability is usually defined as an unexpected, specific, and persistent failure to acquire efficient academic skills despite conventional instruction and adequate intelligence and sociocultural opportunity.[3] Although all fields can be affected, it seems that learning problems can be classified into two major categories. The largest group of children has problems with language skills, i.e., in reading and spelling; this is the dyslexia group with the basic phonologic processing disabilities. The other group of children has relatively greater problems with problem solving, with arithmetic and visuospatial problems, and with motor and tactile perceptual problems. These children are form the large group of those with nonverbal learning disabilities. It comprises the children with dyscalculia. Perhaps one of the most intriguing observations is that children in both groups also show cognitive strengths. Many children in the dyslexia group show relative proficiencies in problem solving and in visuospatial skills. These two features highlight the normal intelligence in most dyslexic children and their artistic talents (to be discussed later).

The disorder with specific difficulties in reading was identified by Oswald Berkhan in 1881[4] and the term ‘dyslexia’ was coined in 1887 by Rudolf Berlin, a German ophthalmologist practicing in Stuttgart, Germany.[5] He used the term to refer to a case of a young boy who had a severe impairment in learning to read and write in spite of showing normal intellectual and physical abilities in all other aspects.(http://www.Wikipedia.org).

The advent of neuroimaging techniques to study brain structure and function enhanced the research in the 1980s and 1990s. Since then, interest has largely been on the neurological basis of this disorder. Current models of the relation between the brain and dyslexia generally focus on some form of defective or delayed maturation of brain. More recently, genetic research has provided increasing evidence supporting a genetic origin for dyslexia. Researchers are searching for a link between the neurological and genetic findings and this reading disorder. There are many previous and current theories of dyslexia; one that has much support from research is that whatever the biological cause, dyslexia is a matter of reduced phonological awareness–the ability to analyze and line units of spoken and written languages.[6] A study from Hong Kong, using functional magnetic resonance imaging (fMRI) has recently shown that children reading English (an alphabetic language) used a different part of the brain than those reading Chinese (a nonalphabetic language).[7] Positron emission tomography (PET) and fMRI studies have produced clear evidence of structural differences in the brains of children with reading difficulties from that in normal children. Dyslexic children have a deficit in parts of the dominant hemisphere (usually the left) in the inferior frontal gyrus, inferior parietal lobule, and middle and ventral temporal cortices.[8,9] Furthermore, autopsy and neuroimaging studies suggest that dyslexic brains are usually symmetric compared to the assymetric brains in normal children,[10] in whom a large left hemisphere (dominant hemisphere) in seen. Earlier, microscopic studies revealed the presence of ectopias and microgyria in the inferior frontal and temporal cortices of dyslexic brains.[11] Although all children with dyslexia do not have these changes, these studies suggest some anatomical correlation with the functional disability.[12]

The dyslexic patient's writing is characterized by spelling errors, letter order errors, letter addition/subtraction, and a small written vocabulary. The writing speed is slow; the hand writing poor, with irregularly formed letters; and inappropriate use of words is common. Reading passages written by these patients is at times is a near impossibility since they often use highly phonetised spelling (e.g., ‘shud’ for ‘should’) and have difficulty in distinguishing between homophones (‘their’ and ‘there’).

Dyslexics, occasionally have poor motor skills, with poor coordination and clumsiness. Dyslexia, as indicated earlier, should not be confused with dyscalculia; in fact, dyslexic individuals can often be gifted in mathematics (e.g., Albert Einstein). However, descriptive mathematics and engineering or physics problems that rely on written texts rather than numbers may pose problem (exceptions were Leonardo da Vinci and Einstein). Also, there may be difficulty remembering mathematical facts, such as multiplication tables, and in learning the sequence of steps when performing calculations. Response to mathematics drills is often slow.

It is important to realize at this stage that the disability in learning disorders is often domain specific (just like intelligence) and all affected individuals may not manifest all of the features detailed above. Specific brain regions or specific circuitry performs specific functions, the details of which have not yet been unraveled.

## Artistic Creativity and Dyslexia

Dyslexic people often have a natural flair for one or more of the arts (such as music, dance, drawing, or acting). They often possess a natural ability to see patterns in noise, which helps them to produce creative abstract ideas out of what many would look upon as mundane sensory environments.[13]

Leonardo da Vinci (1452-1519) is one of the greatest creative artists the world has ever seen. He painted such masterpieces as the Mona Lisa, The Madonna and Child, Leda and the Swan, and had also made detailed drawings of human anatomy. In addition, he excelled as an engineer (he planned and sketched an entire canal system in ancient Italy and designed bridges) and architect (drawing architectural plan of churches). From a detailed study of his writing it has been suggested that Leonardo da Vinci might have had dyslexia.[14] Back in 1977, Cohn and Neumann[13] in a study of dyslexic children, observed that some have an outstanding ability to produce artistic pictures and objects. These productions were perceptive, well organized, and generally contained much action. Despite these pictorial skills, these patients might show only a rudimentary use of coded symbolic graphic forms. These workers postulated for the first time that artistic production is generated by the nondominant hemisphere and that this function is quite distinct from the coded graphic operation resident in the dominant hemisphere (this will be discussed later in this article). In a more recent study,[15] it has been found that art academy students reported significantly more signs of dyslexia than non-art university students. Objective testing showed poorer phonological skills among art students. The prevalence of dyslexia was far higher among art students.

## Creativity in Dyslexia – Lesson from Einstein's brain

Albert Einstein (1879-1955) is undoubtedly one of the greatest creative geniuses of modern times. As a child, he started talking late and he possibly had developmental dyslexia. Einstein's own description[16] of his scientific thinking was that ‘…words do not seem to play any role,’ but there is ‘associative play’ of ‘more of less clear images’ of a ‘visual and muscular ideation.’ A detailed study of his brain was carried out as per his will. Macroscopically, the exceptional finding was that, in Einstein's brain, the stem of the lateral sulcus was found to be continuous with the bottom end of the postcentral sulcus.[17] This is in contrast to the usual anatomy, where the posterior ramus of the lateral sulcus goes beyond the postcentral sulcus into the parietal lobe on which lies the supramarginal gyrus; the angular gyrus lies behind it and the two constitute the Wernicke area. These two gyri together with the part of the parietal lobe lying below the intraparietal sulcus constitute the inferior parietal lobule corresponding to Broadman areas 39 and 40. The peculiar anatomical variation in Einstein's brain resulted in a large undivided inferior parietal lobule (areas 39 and 40). The inferior parietal lobule, specially in the nondominant hemisphere is the seat of visuospatial cognition, mathematical ideation, and imagery movement, as also of artistic and literal creativity.[18,19] In Einstein's brain, as a result of the altered sulcal anatomy, this area was larger than that in controls and, in addition, was aslo undivided, allowing for uninterrupted connectivity. Histological study by Diamond and others[20] earlier indicated that Einstein's area 39 showed a lesser number of neurons but proportionately a higher number of glial cells. This was later interpreted as suggestive of greater connectivity.[21] Creative and mathematical genius of Einstein thus seemed related to his large undivided inferior parietal lobule (areas 39 and 40) and greater connectivity in this region. In 1985, Geschaind and Galaburda[22] posited that delay in development of the left hemisphere (dominant) may allow the right hemisphere (non-dominant) which mediates spatial computing to become highly specialized.[21–23] It can thus be postulated that Einstein's developmental dyslexia, causally related to a developmental delay of the dominant hemisphere, might have led to the non-dominant hemisphere being highly specialized for spatial computation. This hypothesis seems a sound one but difficult to prove and fail to explain why all dyslexic children do not become as creative as Einstein. Neurological outcome from localized brain injury is extremely individualized and depends a lot on plasticity and connectivity.

## Artistic Creativity and Neurological Disorders

Like visuospatial cognition, artistic skills most likely rests in the parietal lobe (specially the nondominant one) and more so in the inferior parietal lobar region[18]. Some of the recent observations indicate that the dominant hemisphere has an inhibitory influence on creative (especially artistic and literary) functions of the nondominant parietal lobe[19]. Does that mean that ‘disinhibition’ of the nondominant parietal lobe from a dominant hemisphere lesion would increase artistic and literary creativity?

There are several reports of *de novo* development of artistic behavior following brain injury, especially injury affecting the dominant hemisphere.[23] Such pathologies include a left temporal lobe epileptic focus, dominant hemisphere stroke, subarachnoid hemorrhage caused by middle cerebral aneurysm and, most importantly, frontotemporal dementia (where dominant hemisphere affection with language disorder is well recognized). The case histories of such patients support the notion that a dominant hemispheric dysfunction may lead to ‘disinhibited’ functioning of the nondominant parietal lobe, thereby unmasking hidden artistic talent, a phenomenon described as ‘paradoxical functional facilitation.’[24]

## Artistic talent in Dyslexia – A hypothesis

Based on the arguments relating creativity to dyslexia, as in Einstein's case and *de novo* development of artistic creativity in patients with dominant hemisphere brain injury, it can be hypothesized at this stage that the unmasking or development of artistic talents observed in many dyslexic subjects may be linked to the developmental delay in language function, which localized in the dominant hemisphere (usually the left hemisphere). This would ‘disinhibit’ the nondominant parietal lobe (usually the right), leading to unmasking or development of artistic talent and creativity. Creativity, of course, is domain specific, and it need not be limited only in to the field of art.

In support of this hypothesis that underfunctioning of one part of the brain may lead to disinhibited functioning of another part and unmasking of a skill or talent, are the cases where artistic talents have been recognized in children with autism spectrum disorders.[25] which fundamentally result from a developmental underconnectivity in the brain.[26] Such talented autistic patients are often referred to as autistic savants.[27,28] The literal meaning of the word ‘savant’ is ‘scholar.’ I feel that dyslexic children with exceptional artistic or other talents may also be referred to as ‘dyslexic savants.’

## Neurology in *Taare Zameen Par*

By now it should be clear to readers what my focus is. In the film, *Taare Zameen Par*, the child Isham Aswasthi is ridiculed at school and at home for not being able to write and read properly. He has developmental dyslexia. He seems to be of average intelligence and his spontaneous speech is fluent and meaningful. From the very beginning of the film, his facial expression appears very innocent, but it is tinged with stupidity. In fact, though he is ‘stupid,’ he has the intelligence to get his letter requesting absence from school written by his elder brother. Isham cannot read, and his writing contains several spelling mistakes and the use of inappropriate letters and words. He is frustrated and this is evident through his behavior at times (kicking flower pots) but he has no other conduct disorder. His apparent hyperactivity (shown in a scene of his taking a shower) is perhaps a normal phenomenon in children of his age. However, he certainly lacks motor skills (as is known to occur in dyslexia) as he cannot knot his tie or his tie shoelaces properly. His inability to perform simple arithmetic is a bit of a puzzle; this is unusual in dyslexia. Isham does not have dyscalculia, he has a prominent language deficit. Perhaps this has been portrayed to enhance the image of helplessness and disability in this young boy. At times the boy looks vacant, absorbed in his own thoughts. Such behaviors (daydreaming, perhaps) are not unusual in his age and such cases are often referred to neurologists as possible absence seizure spells. The film has a happy ending, however, with Ishan's artistic talent being discovered by his art teacher Ramshankar Nikumbh. In fact, his art teacher makes the diagnosis of dyslexia after examining Ishan's class note books. The discovery of Ishan's artistic talent and the way it blooms under the care of his art teacher is the most important (and joyous) neurocognitive phenomenon in the film. Ishan's case, seems to be related to the phenomenon of ‘disinhibition’ of his right parietal lobe (Ishan is right-handed and hence his right parietal must be the nondominant one), which is related in turn to the developmental delay of his left hemisphere (the dominant hemisphere) that is manifested in his developmental dyslexia. Ishan Awathi may thus be labeled as an example of ‘dyslexic savant syndrome.’ Furthermore, the improvement in his artistic skill triggered by the support of his art teacher (Ishan went on to receive the first prize in the school painting competition) highlights a major and therapeutically important issue in cognitive neuroscience and neuropsychology — namely cosmetic neurology.[28]

## Concluding Remarks

In remedial training in children with dyslexia, it is important to facilitate the development of their unique artistic and other abilities to its full capacity rather than to overemphasize on the correction of the disturbed coded symbol operations. This film should be an eyeopener to parents of dyslexic children. The entire team and in particular the young artist, Darsheel, who so vividly portrays the helplessness of a disabled child, deserve our appreciation.
